# Pediatric Multi-Organ Dysfunction Syndrome: Analysis by an Untargeted “Shotgun” Lipidomic Approach Reveals Low-Abundance Plasma Phospholipids and Dynamic Recovery over 8-Day Period, a Single-Center Observational Study

**DOI:** 10.3390/nu13030774

**Published:** 2021-02-27

**Authors:** Mara L. Leimanis-Laurens, Karen Ferguson, Emily Wolfrum, Brian Boville, Dominic Sanfilippo, Todd A. Lydic, Jeremy W. Prokop, Surender Rajasekaran

**Affiliations:** 1 Pediatric Critical Care Unit, Helen DeVos Children’s Hospital, 100 Michigan Street NE, Grand Rapids, MI 49503, USA; karen.ferguson@spectrumhealth.org (K.F.); brian.boville@helendevoschildrens.org (B.B.); dominic.sanfilippo@helendevoschildrens.org (D.S.); surender.rajasekaran@spectrumhealth.org (S.R.); 2 Department of Pediatric and Human Development, College of Human Medicine, Michigan State University, Life Sciences Bldg. 1355 Bogue Street, East Lansing, MI 48824, USA; prokopje@msu.edu; 3 Van Andel Institute, Bioinformatics & Biostatistics Core, 333 Bostwick Avenue NE, Grand Rapids, MI 49503, USA; Emily.Wolfrum@vai.org; 4 Department of Physiology, Collaborative Mass Spectrometry Core, 567 Wilson Road, East Lansing, MI 48824, USA; lydictod@msu.edu; 5 Department of Pharmacology and Toxicology, Michigan State University, 1355 Bogue Street, East Lansing, MI 48824, USA

**Keywords:** lipidomics, pediatrics, critical illness, multi-organ dysfunction syndrome, phospholipids, nutritional intake

## Abstract

Lipids are molecules involved in metabolism and inflammation. This study investigates the plasma lipidome for markers of severity and nutritional status in critically ill children. Children with multi-organ dysfunction syndrome (MODS) (*n* = 24) are analyzed at three time-points and cross-referenced to sedation controls (*n* = 4) for a total of *N* = 28. Eight of the patients with MODS, needed veno-arterial extracorporeal membrane oxygenation (VA ECMO) support to survive. Blood plasma lipid profiles are quantified by nano-electrospray (nESI), direct infusion high resolution/accurate mass spectrometry (MS), and tandem mass spectrometry (MS/MS), and compared to nutritional profiles and pediatric logistic organ dysfunction (PELOD) scores. Our results show that PELOD scores were not significantly different between MODS and ECMO cases across time-points (*p* = 0.66). Lipid profiling provides stratification between sedation controls and all MODS patients for total lysophosphatidylserine (lysoPS) (*p*-value = 0.004), total phosphatidylserine (PS) (*p*-value = 0.015), and total ether-linked phosphatidylethanolamine (ether-PE) (*p*-value = 0.03) after adjusting for sex and age. Nutrition intake over time did not correlate with changes in lipid profiles, as measured by caloric and protein intake. Lipid measurement in the intensive care environment shows dynamic changes over an 8-day pediatric intensive care unit (PICU) course, suggesting novel metabolic indicators for defining critically ill children.

## 1. Introduction

It has been estimated that twenty percent of critically ill patients present to the pediatric intensive care unit (PICU) with multi-organ dysfunction syndrome (MODS) [1]. This group experiences ten times the mortality rate compared to other PICU patients [2]. In this group, there exists a smaller cohort of patients who require aggressive life support measures, such as extracorporeal membrane oxygenation (ECMO), with up to 45% reported mortality [3]. The reasons for this are unclear. Understanding MODS has become increasingly important because COVID-19 patients who present with MODS experience a more protracted ICU course and suffer higher mortality than those who have single system involvement [4,5].

Critically ill children experience a hypermetabolic stress response and catabolic state [6]. During this acute illness phase, a rapid breakdown of adipose tissue occurs, leading to multiple sequelae, including chronic inflammation, hyperglycemia, and further damage to organ function by fatty infiltration [7,8]. These patients have often ceased oral intake, thus, being deprived of optimal nutritional support [9,10]. Critical illness by itself is characterized by mitochondrial dysfunction [11] and perturbations in β-oxidation [12] from organ failure. MODS results from various triggers, both infectious and non-infectious, that results in a common phenotype of organ dysfunction. How lipid levels and composition vary over time and between patients with MODS alone verses. those progressing to circulatory collapse and the need for ECMO remains unknown. This knowledge could enable optimization of nutrition therapy, as well as identify new targets for supporting organ function in the face of critical illness.

Unlike some biological molecules, lipids are abundant, making them attractive metabolites to study in MODS patients admitted to the PICU. The role of lipids in critical illness and acute inflammation has been explored in adults [13] and children [14], and lipids are crucial for membrane formation, signaling, and metabolism [15,16,17,18,19]. It is well known that disruption of lipid signaling pathways and metabolism leads to inflammatory disorders [20]. This can be amplified when exogenous lipid sources from the diet are altered, such as in acute illness leading to malnutrition and poor outcomes [21]. Specifically, phospholipids are known to play a role in a wide array of diseases (see Reference [22]). Thus, lipids likely serve as markers for tissue injury, inflammation, metabolic dysfunction, and indicators of nutritional status.

A two time-point lipidomics study focusing on single organ injury in adult patients with acute respiratory distress syndrome (ARDS) revealed 90 significantly different lipids that distinguished survivors from non-survivors [13]. We hypothesize that certain lipid classes can be used to distinguish patients with MODS that need ECMO from those that recover with just medical management accounting for both severity and nutritional status. Advances in the availability and ease of use of lipid profiling in a clinical setting develop signatures of highly personalized lipidome profile to be developed to identify patients experiencing a severity of illness needing ECMO [23], and such a strategy has the potential to be more robust than the rise or fall of a single biomarker. This study uses an unbiased untargeted lipidomics approach to enable a comprehensive study of pediatric lipid profiles, determining the molecular compositions of analytes in blood plasma using direct infusion high resolution/accurate mass spectrometry (MS) and tandem mass spectrometry (MS/MS).

## 2. Materials and Methods 

### 2.1. Study Population, Site, and Sample Collection

Patients who were critically ill with MODS were recruited from the PICU at Helen DeVos Children’s Hospital (HDVCH), a quaternary care facility in Western Michigan, after screening for eligibility and subsequently consented. The HDVCH PICU, with over 1500 admissions per year, and over 6000 patient days, covers a 24-bed unit. Samples were collected at up to three independent time-points: Baseline, >48 h, and >7 days, if they remained patients of the PICU. If a patient was discharged or passed away, no further samples were collected. All patients consented to the study recruitment, as per local Institutional Review Board approval (2016-062-SH/HDVCH) [24].

**Figure 1:**
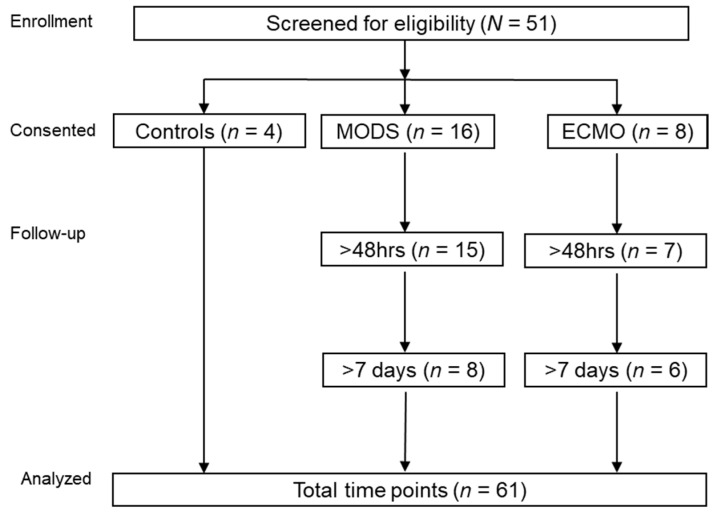
Flow diagram of the patient in the study (*N* = 28). MODS, multi-organ dysfunction syndrome; ECMO, extracorporeal membrane oxygenation.

Patients were referred by the attending physician on service once MODS was recognized, according to the following inclusion criteria: <18 years of age; on vasopressors with a central line and requiring invasive mechanical ventilation for respiratory failure as per criteria established by Proulx et al. [25]. Patients presenting for routine sedation were used as controls. Patients were excluded if they had a diagnosed autoimmune disease, were considering non-interventional treatment options, had undergone cardiopulmonary bypass prior to the onset of MODS, received plasmapheresis prior to ECMO initiation, or were patients of the neonatal intensive care unit at the time of consent. Blood samples were drawn in EDTA-treated tubes, centrifuged, plasma was separated, and stored at −80 °C. In the case of ECMO patients, samples were drawn right before going on the circuit with a median of three days into their PICU admission (Figure 1—Study Flow Chart).

### 2.2. Data Collection

Basic demographic variables were extracted from the local electronic medical record (EMR). Dietary history, and mode of feeding [nil per os (NPO), per os (PO), tube feeding (TF) ± lipids, total parenteral nutrition (TPN) ± lipids] was extracted from the dietician’s notes in the EMR. Percent calories and protein were calculated from the resting energy expenditure [26] and daily required intake [27]. They were considered qualified according to less than or equal to 33% of needs met, 34–66%, and greater or equal to 67% nutritional needs met. Mode of respiratory support was qualified according to mechanical ventilation (MV), nasal cannula (NC), or room air (RA). All study data were collected and managed using REDCap [28]. The severity of illness scores were retrieved through the Virtual Pediatric Intensive Care Unit Performance Systems (VPS, LLC, Los Angeles, CA, USA). PELOD was included, given that it is a measure of the first 10 days in the ICU. The median time to consenting patients was two days from ICU admission, and Pediatric Index of Mortality 2 (PIM2) and Pediatric Risk of Mortality III (PRISMIII) were both calculated during the first hours of ICU admission only, and therefore, not included in our study analysis.

### 2.3. Blood Plasma Lipidomics Method

Blood samples collected in EDTA-treated tubes were immediately placed on ice and then spun at 4 °C (once for 15 min at 1500 rpm; a second spin for 10 min at 10,000 rpm), plasma was harvested and frozen to −20 °C, and −80 °C for long-term storage. Lipidome profiles were determined from 5 µL of plasma thawed on ice, while the remainder of the plasma sample was reserved for other studies. Each 5 µL aliquot was diluted in 95 µL of HPLC-grade water and subjected to lipid extraction in 2 mL glass tubes with PTFE-faced caps, using an extraction mixture of acetone, methanol, and acetonitrile (1:1:1, *v:v:v*) according to [29]. Lipid extracts were dried under nitrogen and reconstituted in isopropanol:methanol:chloroform (4:2:1, *v:v:v*) by gentle vortexing for 1 min. Di-myristoyl phosphatidylcholine was spiked into each sample during extraction as an internal standard, such that the final concentration was 0.5 pmol/µL in the reconstituted lipid extracts. Immediately prior to mass spectrometric analysis, aliquots of each plasma lipid extract were loaded into an Eppendorf 96 well plate and evaporated under nitrogen. The lipids were then resuspended in a solvent of 20 mM ammonium formate in isopropanol:methanol:chloroform (4:2:1, *v:v:v*), and the 96 well plate was sealed with a sealing mat (Analytical Sales and Services). The 96 well plate was then loaded into an Advion Nanomate Triversa (Advion Biosciences, Ithaca, NY, USA) that served as the nano-electrospray ionization source and high-throughput autosampler. The autosampler temperature was held at 12 degrees C during the analysis. The Nanomate spray voltage was held at 1.4 kV and a gas pressure of 0.3 psi. Under these conditions, the Nanomate operates at an nESI flow rate of approximately 500 nL per minute. Five microliters of each lipid extract was directly infused into an LTQ-Orbitrap Velos mass spectrometer (Thermo Scientific, Waltham, MA, USA) with the FT analyzer operating at 100,000 resolving power (defined at *m*/*z* 400) and a scan rate of 1 Hz. Full MS scans were collected for one minute each in positive and negative ionization modes. The inlet of the mass spectrometer was held at 100 degrees C, the S-lens was set to 50 percent, and the trap accumulation time was 300 milliseconds. Under these conditions, in-source fragmentation is minimal under nESI conditions. To verify identities of abundant lipids, ion mapping MS/MS was performed on pooled lipid extracts using higher-energy collisional dissociation at a normalized collision energy of 60 and 100,000 resolving power, at a step size of 1.0 mass units between *m*/*z* 200 and 1000, and a trap accumulation time of 1000 milliseconds. Prior to MS data collection, mass calibration was performed on the FT analyzer according to the vendor’s instructions using an automated calibration routine. Following initial data collection, each mass spectrum was additionally subjected to offline mass recalibration using the Xcalibur software (ThermoFisher Scientific, USA) Recal Offline tool in order to further refine mass accuracy and eliminate drift in instrument calibration over the duration of the analytical run. The peak findings, correction of ^13^C isotope effects, and quantification for global lipidomics were performed with Lipid Mass Spectrum Analysis (LIMSA) version 1.0 software [30] as previously described [31]. The software vendor’s “linear fit” algorithm was used for isotope correction, and a mass search window of 0.003 *m*/*z* was utilized to match MS1 peaks to known endogenous lipids and the spiked synthetic internal standard. All calculated peak areas of found peaks were normalized to that of the internal standard. Due to the untargeted nature of the analysis, no attempts were made to quantitatively correct for differences in ionization efficiencies across lipid species owing to length and degrees of unsaturation of the lipid acyl chains or the polarities of lipid headgroups. All quantitated found lipid peak data from separate positive and negative ion analyses were subsequently combined in Microsoft Excel software for the purpose of downstream data analysis and statistical evaluation. Lipidome analysis provided untargeted assessment across all classes of glycerolipids (GC) (including mono-, di- and triglycerides), phospholipids (PS), lysophospholipids (lysoPL), sphingolipids (SP), sterols, non-esterified fatty acids (NEFA’s), and fatty acids (FAs). Additional species analysis was completed on phospholipids (PL), triacylglycerides (TGs), diacylglycerides (DGs), cholesterol (chol), and sphingomyelins (SMs). Large blood volumes from this patient population were challenging to obtain, therefore, plasma volumes were small (~0.050–0.075 mL total). As only a small fraction of each sample was available for lipidomic analysis, primarily higher abundance lipids were targeted [32].

### 2.4. Analysis

Percent data were transformed before being analyzed with a beta regression from the R [33] package betareg [34]. Total normalized ion values were log-transformed and analyzed using generalized linear regression models (glm) in R [35]. All regression models were adjusted for age and sex. Contrasts between treatment groups (sick vs. sedation) were conducted using R package emmeans [36]. Correlation analysis comparing percent phospholipids with caloric and protein intake (from Supplemental Table S2) over three time-points was completed using generalized linear models with a logit link, adjusted for age and sex, and stratified by time. Partial R^2^ for percent caloric intake and percent protein intake, respectively, were calculated from the regression output using the rsq package [37]. *P*-values from the regression models have been corrected for multiple testing via the FDR method. Additional statistical tests that were performed include Welch’s *t*-tests, independent *t*-tests, and Wilcoxon Rank Sum tests. The *p*-values from these tests were not multiple testing corrected. Lipid analysis was done on all major lipid classes (as stated above). In this paper, we focused on phospholipids, given they are heavily influenced by exogenous dietary sources.

## 3. Results

### 3.1. Study Population

Basic characteristics revealed a majority of male (60.6%) Caucasian (57.1%) patients (Table 1). Ages ranged from neonates to adolescents (0.14–202 months) with median values of 94.25 months for control patients (range 28.0–122.5), 114.50 months for MODS (range 0.14–202), and 3.5 months for ECMO patients (range 0.5–202), age ranges for both patient groups were similar. Total hospital length of stay (HLOS) ranged between 5–377 days, and total PICU LOS ranged from 3–79 days; ECMO patients spent almost two times as long in the PICU. The majority of patients had a diagnosis of bronchiolitis/pneumonia (7/24; 29%), or sepsis (7/24; 29%). There were two mortalities at one years’ time—both were patients requiring ECMO. All MODS patients at baseline were mechanically ventilated, had their lungs and heart compromised, and were administered inotropes to support cardiovascular function. More than half of the participants exhibited renal dysfunction (*n* = 15; 54%), including seven MODS and all eight ECMO patients (odds ratio: 6.01, *p*-value = 0.01). Other organs affected included liver (*n* = 8; 28.6%; 4 MODS; 4 ECMO) and brain (*n* = 6; 21.4%; 4 MODS; 2 ECMO) in approximately one-quarter (See supplemental Figure S7). The severity of illness scores Pediatric Logistic Organ Dysfunction-2 score (PELOD) was not significantly different between MODS and ECMO groups across time-points (Global F-stat, *p =* 0.66). This suggests that common metrics of severity do not reflect the need for ECMO—further suggesting the need for additional clinical stratifiers, such as assessing nutrition or lipidomics.

### 3.2. Baseline Lipid Profiles of Critically Ill Children Compared to Controls 

**Figure 2:**
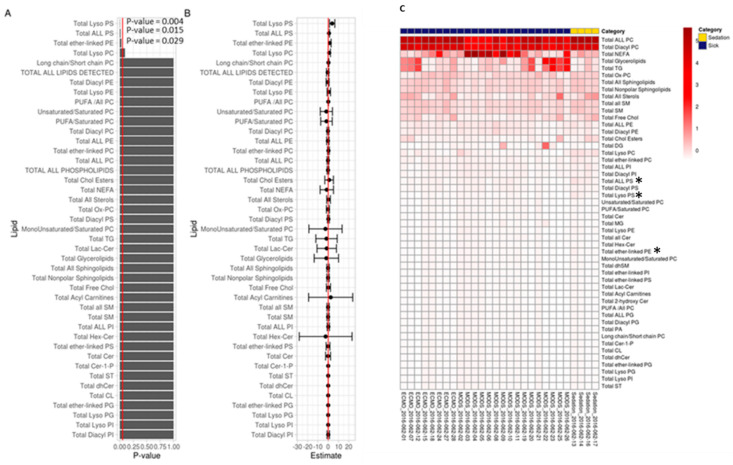
Total Baseline Lipids Based on Absolute Normalized Ion Abundances per ml of Plasma. (**A**)—Bar chart of *p*-values from regression output from log-transformed data; red line indicates 0.05. Any *p*-value that crosses 0.05 is not statistically significant. (**B**)—Points in B represent the mean log(fold-change) in odds between sick and sedation groups. The error bars in B, represent the upper and lower bounds of the false-coverage for the estimate. 95% false coverage intervals of the mean log (fold-change) in odds; red line indicates 0. Any confidence interval that crosses 0 is not statistically significant. (**C**)—Scaled values of total lipids based on absolute normalized ion abundances per ml of plasma; * Statistically significant values (*p*-value ≤ 0.05), applied to those values from A and B of statistical significance. Lipid categories according to Quehenberger et al. [38]: Glycerophospholipids (*n* = 23); Fatty acids (*n* = 1); Sterol lipids (*n* = 3); Glycerolipid (*n* = 2); Sphingolipid (*n* = 7); Acyl carnitine (*n* = 1). Abbreviations: Cer, ceramide; Chol, cholesterol; CL, cardiolipin; dhCer, dihydrosphingomyelin; Hex-Cer, hexosylceramide; Lac-Cer, lactosylceramides; NEFA, non-esterified fatty acid; P, phosphate; PC, phosphatidylcholine; PE, phosphatidylethanolamine; PG, phosphatidylglycerol; PI, phosphatidylinositol; PS, phosphatidylserine; PUFA, polyunsaturated fatty acid; SM, sphingomyelin; SP, sphingolipid; ST, sterols; TG, triglycerides.

In order to determine the source of differences in lipid classes at baseline, when most patients (67%) were NPO, we examined absolute normalized abundance (per mL of plasma, Figure 2). Lipids in MODS patients (ECMO and MODS combined) differed significantly at baseline from sedation control samples after correcting for multiple testing based on generalized linear models adjusted for age and sex for three classes of phospholipids: Total lysophosphatidylserine (lysoPS) (*p*-value = 0.004), total phosphatidylserine (PS) (*p*-value = 0.015), and total ether-linked phosphatidylethanolamine (ether-PE) (*p*-value = 0.03) (Figure 2A). Point estimates and confidence intervals of the differences are presented in Figure 2B. The heatmap revealed relative baseline levels for all lipid classes analyzed, including NEFAs, glycerolipids, and TGs (Figure 2C). Lipid species were evaluated, no significant differences were found (Supplemental Figures S1–S5).

NEFA levels were further explored and found to be significantly associated with sex. However, after adjusting for sex, none of the experimental groups were significantly associated with NEFA levels at any time-point.

To serve as an additional internal control, total plasma serine (*O*-acetyl-l-serine) levels were compared between the three groups, from a metabolite dataset on the same patient cohort (to be presented in a separate report). Mean values were not significantly different (sedation vs MODS; *p* = 0.8281; sedation vs. ECMO; *p* = 0.3348) based on independent *t*-test. This may imply that in spite of plasma serine levels, the PS and lysoPS values differ for all MODS patients, due to other metabolic drivers.

### 3.3. Mode of Feeding and Nutritional Intake

With detailed notes of nutrition for control and MODS patients, the data can be used to investigate feeding and nutritional intake to elucidate biases in the cohort and to qualitatively control for intake into the lipidomic analyses below. Mode of feeding and both percent and total caloric and protein intake were reviewed for each patient (Table 2; Supplemental Tables S1 and S2). Sedation controls were NPO (nil per os, nothing by mouth) for eight hours prior to the procedure. At baseline, 16/24 (67%) of all combined MODS (MODS + MODS who required ECMO) patients were NPO, which decreased (to 7%) by day 8. Baseline caloric intake was less than 33% of the recommended intake for most patients 19/24 (79%), which gradually improved over time. A similar profile was reflected in the percent total protein. ECMO patients by eight days were achieving higher percent calories and protein than their MODS counterparts (over 34% goal reached): ECMO (*n* = 5; 83%) vs. MODS (*n* = 4; 50%) and ECMO (*n* = 6; 100%) vs. MODS (*n* = 4; 50%), respectively. There was no evidence of a statistical difference in the percentages of protein or total calories when tested with Fisher’s exact test (*p* = 0.085; *p* = 0.30). All patients were experiencing a deficit nutritionally, however ECMO patients were reaching superior nutritional intake, which may be due to their being more hemodynamically stable because of the circulatory support, better facilitating their caloric need. Of further importance is how this exogenous nutritional intake influences blood plasma lipid profiles for patients over time. Based on this result, we might anticipate that ECMO patients fare better than MODS in terms of overall lipid profiles.

### 3.4. Phospholipids of Critically Ill Children over Three Time-Points

**Figure 3:**
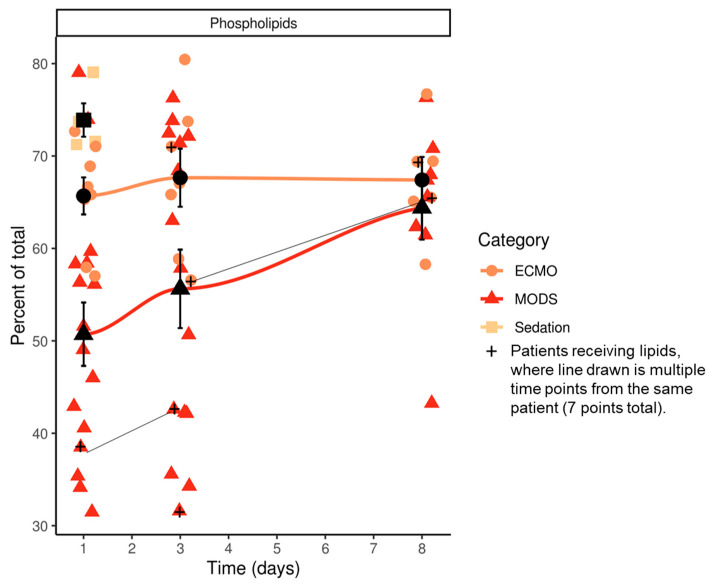
Time course for lipids over eight days. ECMO, extracorporeal membrane oxygenation; MODS, multi-organ dysfunction syndrome.

The phospholipid distributions (percent total) of MODS patients who needed ECMO, and patients coming to the hospital for same-day sedation are illustrated in Figure 3 over three time-points, connecting the group mean at each time-point. Phospholipid levels increase over time which did not correlate with percent nutritional intake (caloric: baseline *R* = 0.13; 72 h *R* = −0.04; 8 days *R* = 0.02 and protein: *R* = 0.13, 72 h R = 0.00; 8 days *R* = 0.01) (Table 2; Supplemental Figure S6). A total of five patients (*n* = 2 MODS; *n* = 3 ECMO) over seven time-points received IV lipids as a portion of their nutritional regimen. Two patients received lipids at more than one time-point, both with slight increases in their plasma phospholipid levels.

These differences may not be based on nutrition intake alone, given most patients were NPO (67%) at baseline, and receiving less than 33% of needed intake (79%), suggesting other metabolic drivers at play.

Figure 3 shows the raw lipid values for each patient across time. Groups are differentiated by color and by shape. The lines connect the group means at each time-point. The black shape and error bars indicate the group mean ± SE.

In summary, we see down-regulated phospholipid levels, including sub-classes (lyso PS, PS, ether-PE) at baseline for patients with MODS, with increases by day 8, except for those with MODS that needed ECMO (Figure 2 and Figure 3). Increases in plasma phospholipids did not correspond to nutritional intake for these patients.

## 4. Discussion

Patients with MODS present with dyslipidemia at the first detection of organ pathology based on our lipidomic profiles presented in this report. Patients with MODS are compromised at the time of baseline, undergoing membrane disruption/remodeling, catabolic state, cannibalism, and lipid dysregulation [39], resulting in decreased phospholipids—as has been previously reported for pneumonia [40] and septic patients [41]. Some reports state that phospholipids from endogenous sources, such as the hepatobiliary system [42], are involved with a signaling of the innate immune response [43]. In the event of multi-organ dysfunction, with liver involvement (as we see in half of our ECMO patients), perhaps this is not entirely surprising. We suspect that this observed difference in ECMO patients may be tied to severe cardiac dysfunction with an inability to compensate metabolically. Generally, risk factors for this sub-population may include pre-existing conditions, such as un-diagnosed conditions, metabolic syndrome, or other organ pathologies, which have been documented in COVID-19 cases [44], as reported by other groups. 

Recent studies by other groups in COVID 19 infections have found that patients who suffer from multi-organ involvement are more prone to mortality [45]. A subclass of phospholipids, phosphatidylserine was found to be among the lowest-abundance lipids in COVID patients and had predictive power for fatal groups by ROC curve analysis. An untargeted lipidomics approach was adopted for COVID-19 patients, which revealed lower levels of certain classes of lipids that persisted even with a regular diet and discharge home [46]. This finding is indicative of ongoing metabolic disruption, post-ICU admission. 

Specifically, a sub-class of lyso lipids, known as lysophosphatidylserine (lysoPS), has immune function [47,48] through enhanced clearance of neutrophils [49]. Synthesized by neutrophils [50,51,52], lysoPS enhances efferocytosis (“to carry to the grave” [53]) of neutrophils by macrophages [54] during acute inflammation [55,56]. Neutrophil levels were lower and more like controls in patients who needed ECMO [57], and other studies have shown that neutrophil levels drop in the sickest patients as bone marrow activity becomes suppressed. This may lead to lower levels of lysoPS.

Over the past decades, the externalization of PS has been linked with efferocytosis, and characterized as the ultimate “eat-me” signal, which is an evolutionarily conserved immunosuppressive signal, which prevents local and systemic immune activation [58]. This externalization of PS has been shown to be exploited by viruses, microorganisms, and parasites to promote infection [58]. This could link PS levels seen in our cohort to infection in our cohort as many of our patients presented with bronchiolitis, pneumonia, and sepsis, two of which had Coronavirus (OC43 and HKU1). Many of the patients in this study had viral illness as a trigger or in addition to MODS, as is typical in the PICUs across the country.

Untargeted lipidomic research was done previously in adult patients (*n* = 30) with ARDS using a shotgun lipidomics approach revealed 90 significantly different lipids that distinguished survivors from non-survivors [13]. Similarly, in children, it has been shown that profiling metabolites for septic shock and systemic inflammatory response syndrome (SIRS) can yield markers of mortality [23].

In all these patients, the lipidome is reflective of an amalgam of multiple pathways that include the inflammatory cascade, immune dysfunction, severity of illness, and nutritional status, all of which could combine to affect the clinical course. Age was accounted for in the models (given ECMO patients were younger than the MODS), as was suspected that immature immunity or active suppression of the immune system and a confounder [59]. ECMO patients may have their ability to modify their lipidome impeded as the concentrations of major lipid classes are closer to sedation controls. One limitation is the non-standard re-initiation of nutritional support in this patient population, which reflects the observational research experience at the bedside, and provides hypotheses for future research.

## 5. Conclusions

In this prospective observational study, we studied several lipid classes at multiple time-points and identified that phospholipids showed differences between all MODS and sedation, using a shotgun lipidomics approach. Future work may include a targeted lipidomic approach on phospholipids. Patient lipid profiles were not statistically significantly correlated with nutritional interventions over an 8-day period of the PICU stay.

## Figures and Tables

**Table 1:** Patient characteristics of MODS/ECMO Study 2016–2018 (*N* = 28).

Clinical Variables	Range	n	% of Total	Median	IQR	Wilcoxon (All MODS vs. Sedation)	*p*-Value (All MODS vs. Sedation)	Logistic Regression (ECMO vs. MODS)	*p*-Value (ECMO vs. MODS)
Demographics									
Age (months)		28				45.00	0.87		
Controls	28.0–122.5	4	14.3	94.25	31.50 ^β^				
MODS	0.14–202	16	57.1	114.50	131.00 ^€^				
ECMO	0.5–202	8	28.6	3.50	84.00 ^£^				
Sex (Female)									
Controls		2	7.0						
MODS		7	25.0						
ECMO		2	7.0						
Ethnicity									
Caucasian/European		16	57.1						
Hispanic		7	25.0						
African American		4	14.3						
Asian/Indian/Pacific		1	3.6						
Diagnosis		24							
Bronchiolitis/ Pneumonia ^a^		7	29.2						
Sepsis ^b^		7	29.2						
Acute kidney injury		2	8.3						
Cardiac ^c^		4	16.7						
Other ^d^		4	16.7						
Organs Affected		24							
Lung		24	100.0					0.17	0.68
Liver		8	33.3					2.33	0.13
MODS (*n* = 16)		4	25.0						
ECMO (*n* = 8)		4	50.0						
Kidney		15	62.5					6.01	0.01
MODS (*n* = 16)		8	50.0						
ECMO (*n* = 8)		7	100.0						
Brain		6	25.0					0.41	0.52
MODS (*n* = 16)		5	31.3						
ECMO (*n* = 8)		1	12.5						
PELOD ^e^		24		20.0	10.25				0.05
MODS		16		12.5	10.25				
ECMO		8		21.5	11.25				
Outcomes									
HLOS (days)						80.00	0.20		
MODS	5.0–82	16	0.57	20.00	27.00				
ECMO	7.0–377	8	0.29	35.00	11.50				
PICU LOS (days)						77.50	0.27		
MODS	3.0–79	16	0.57	16.00	12.00				
ECMO	7.0–377	8	0.29	31.00	13.00				
Mortality			0.00						
MODS		0	0.00						
ECMO		2	0.07						

ECMO, extracorporeal membrane oxygenation; IQR, inter-quartile range; LOS, length of stay; MODS, multiorgan dysfunction syndrome; PICU, pediatric intensive care unit. ^β^ For controls (ages: 89.0, 122.5, 99.5, 28.0); ^€^ MODS (0%—0.14; 25%; 13.00; 50%—114.50; 75%—144.00; 100%—202.00); ^£^ ECMO (0%—0.5; 25%—2.0; 50%—3.5; 75%—86.0; 100%—202.0). ^a^ Bronchiolitis/Pneumonia: Respiratory syncytial virus (*n* = 2); Rhino/enterovirus, coronavirus (*n* = 1); *Klebsiella pneumonia*, *Staph aureus* (*n* = 1); *Pseudomonas entomophila* (*n* = 1); Metapneumovirus (*n* = 1); Rhinoenterovirus (*n* = 1), ^b^ Sepsis: Group A Beta Hemolytic Streptococci (*n* = 1); *Streptococcus pyogenes* (*n* = 2); Coronavirus 229E (*n* = 1); Methicillin-resistant Staphylococcus aureus and Rhino/enterovirus (*n* = 1); Rhino/enterovirus (*n* = 1). ^c^ Includes acute myocarditis (*n* = 3), dilated cardiomyopathy (*n* = 1). ^d^ Tracheitis (*n* = 1), Carbon monoxide poisoning (*n* = 1), Hemophagocytic lymphohistiocytosis (*n* = 1), Febrile infection-related epilepsy (*n* = 1). ^e^ Comprehensive PELOD score; *p =* value, based on Mann-Whitney rank sum test.

**Table 2:** Patient Nutrition for MODS/ECMO Study 2016–2018 (*N* = 24).

	Prior to Admit	Baseline (%)	72 h (%)	8 Days (%)
Mode of feeds				
All	(*n* = 24)	(*n* = 24)	(*n* = 22)	(*n* = 14)
PO	13 (54.2)	0 (0.0)	0 (0.0)	1 (7.1)
TF	8 (33.3)	6 (25.0)	11 (50.0)	9 (64.3)
TF + TPN/Lipids	0 (0.0)	0 (0.0)	1 (4.5)	2 ^b^ (14.3)
TPN/Lipids	0 (0.0)	2 ^a^ (8.3)	5 ^c^ (22.7)	1 (7.1)
NPO	0 (0.0)	16 (66.7)	4 (18.2)	1 ^d^ (7.1)
N/A	3 (12.5)	0 (0.0)	1 (4.5)	0 (0.0)
MODS	(*n* = 16)	(*n* = 16)	(*n* = 15)	(*n* = 8)
PO	9 (56.3)	0 (0.0)	0 (0.0)	1 (12.5)
TF	5 (31.3)	4 (25.0)	9 (60.0)	7 (87.5)
TF + TPN/Lipids	0 (0.0)	0 (0.0)	1 (6.7)	0 (0.0)
TPN/Lipids	0 (0.0)	2 ^a^ (12.5)	2 (13.3)	0 (0.0)
NPO	0 (0.0)	10 (62.5)	2 (13.3)	0 (0.0)
N/A	2 (12.5)	0 (0.0)	1 (6.7)	0 (0.0)
ECMO	(*n* = 8)	(*n* = 8)	(*n* = 7)	(*n* = 6)
PO	4 (50.0)	0 (0.0)	0 (0.0)	0 (0.0)
TF	3 (37.5)	2 (25.0)	2 (28.6)	2 (33.3)
TF + TPN/Lipids	0 (0.0)	0 (0.0)	0 (0.0)	2 ^b^ (33.3)
TPN/Lipids	0 (0.0)	0 (0.0)	3 ^c^ (42.9)	1 (16.7)
NPO	0 (0.0)	6 (75.0)	2 (28.6)	1 (16.7)
N/A	1 (12.5)	0 (0.0)	0 (0.0)	0 (0.0)
% Calories Met				
All	(*n* = 24)	(*n* = 24)	(*n* = 22)	(*n* = 14)
≤33	N/A	19 (79.2)	9 (40.9)	5 (35.7)
34–66	N/A	1 (4.2)	8 (36.4)	6 (42.8)
≥67	N/A	2 (8.3)	4 (18.2)	3 (21.4)
N/A	N/A	2 (8.3)	1 (4.5)	0 (0.0)
MODS	(*n* = 16)	(*n* = 16)	(*n* = 15)	(*n* = 8)
≤33	N/A	12 (75.0)	6 (40.0)	4 (50.0)
34–66	N/A	1 (6.3)	4 (26.7)	3 (37.5)
≥67	N/A	1 (6.3)	4 (26.7)	1 (12.5)
N/A	N/A	2 (12.5)	1 (6.7)	0 (0.0)
ECMO	(*n* = 8)	(*n* = 8)	(*n* = 7)	(*n* = 6)
≤33	N/A	7 (87.5)	3 (42.9)	1 (16.7)
34–66	N/A	0 (0.0)	4 (57.1)	3 (50.0)
≥67	N/A	1 (12.5)	0 (0.0)	2 (33.3)
N/A	N/A	0 (0.0)	0 (0.0)	0 (0.0)
% Protein Met				
All	(*n* = 24)	(*n* = 24)	(*n* = 22)	(*n* = 14)
≤33	N/A	19 (79.2)	9 (40.9)	4(28.6)
34–66	N/A	2 (8.3)	8 (36.4)	4 (28.6)
>67	N/A	1 (4.2)	4 (18.2)	6 (42.9)
N/A	N/A	2 (8.3)	1 (4.5)	0 (0.0)
MODS	(*n* = 16)	(*n* = 16)	(*n* = 15)	(*n* = 8)
≤33	N/A	12 (75.0)	6 (40.0)	4 (50.0)
34–66	N/A	1 (6.3)	5 (33.3)	2 (25.0)
>67	N/A	1 (6.3)	3 (20.0)	2 (25.0)
N/A	N/A	2 (12.5)	1 (6.7)	0 (0.0)
ECMO	(*n* = 8)	(*n* = 8)	(*n* = 7)	(*n* = 6)
<33	N/A	7 (87.5)	3 (42.9)	0 (0.0)
34–66	N/A	1 (12.5)	3 (42.9)	2 (33.3)
<67	N/A	0 (0.0)	1 (14.3)	4 (66.7)
N/A	N/A	0 (0.0)	0 (0.0)	0 (0.0)
Resp. Supp.	(*n* = 24)	(*n* = 24)	(*n* = 22)	(*n* = 14)
MV	0 (0.0)	22 (91.7)	21 (95.5)	11 (78.6)
NC	2 (8.3)	1 (4.2)	0 (0.0)	1 (7.1)
RA	22 (91.7)	1 (4.2)	1 (4.5)	(14.3)
N/A	0 (0.0)	0 (0.0)	0 (0.0)	0 (0.0)

ECMO, extracorporeal membrane oxygenation; MODS, multi-organ dysfunction syndrome; MV, mechanical ventilation; NC, nasal cannula; NPO, nil per os-also used here to mean no nutrition support administered; PO, per os/oral feeds; RA, room air; Resp. Supp, respiratory support; TF, tube feeding; TPN, total parenteral nutrition. ^a^ One patient lipids only; ^b^ One patient TF + TPN, no lipids; ^c^ One patient no lipids; ^d^ Patient NPO, however, qualified in the <33% calories/protein met. N/A, no nutritional assessment.

## Data Availability

Data available upon request.

## Supplementary Materials

The following are available online at https://www.mdpi.com/2072-6643/13/3/774/s1, Table S1. Descriptive Patient Nutrition for MODS/ECMO Study 2016–2018 (N = 24); Table S2. Percent calories and protein for MODS/ECMO Study 2016–2018 (N = 24); Figure S1: Lipid Species: Phosphatidylcholine; Figure S2: Lipid Species: Cholesterol; Figure S3: Lipid Species: Diacylglycerol; Figure S4: Lipid Species: Triacylglycerol; Figure S5: Lipid Species: Sphingomyelins; Figure S6: Boxplots of percent phospholipid values colored by group and observed at all three time-points; Figure S7: Organ failure by grouping.

## Abbreviations

ARDS: acute respiratory distress syndrome; Cer: ceramide; Chol: cholesterol; CL: cardiolipin; dhCer: dihydrosphingomyelin; ECMO: extracorporeal membrane oxygenation; Hex-Cer: hexosylceramide; IV lipids: intravenous lipids; Lac-Cer: lactosylceramides; LIMSA: Lipid Mass Spectrum Analysis; lysoPS: lysophosphatidylserine; LOS: length of stay; MODS: multi-organ dysfunction syndrome; MS: mass spectrometry; MV: mechanical ventilation; NC: nasal cannula; NEFA: non-esterified fatty acid; NPO: nil per os; P: phosphate; PC: phosphatidylcholine; PE: phosphatidylethanolamine; PELOD: PEdiatric Logistic Organ Dysfunction; PG: phosphatidylglycerol; PI: phosphatidylinositol; PICU: pediatric intensive care unit; PO: per os/oral feeds; PS: phosphatidylserine; PUFA: polyunsaturated fatty acid; RA: room air; SIRS: systemic inflammatory response syndrome; SM: sphingomyelin; SP: sphingolipid; ST: sterols; TF: tube feeding; TG: triacylglycerides; TPN: total parenteral nutrition.

## References

[1] Typpo K.V., Petersen N.J., Hallman D.M., Markovitz B.P., Mariscalco M.M. (2009). Day 1 multiple organ dysfunction syndrome is associated with poor functional outcome and mortality in the pediatric intensive care unit. Pediatr. Crit. Care Med..

[2] Typpo K., Watson R.S., Bennett T.D., Farris R.W.D., Spaeder M.C., Petersen N.J., Pediatric Existing Data Analysis (PEDAL) Investigators, Pediatric Acute Lung Injury and Sepsis Investigators (PALISI) Network (2019). Outcomes of Day 1 Multiple Organ Dysfunction Syndrome in the PICU. Pediatr. Crit. Care Med..

[3] Barbaro R.P., Paden M.L., Guner Y.S., Raman L., Ryerson L.M., Alexander P., Nasr V.G., Bembea M.M., Rycus P.T., Thiagarajan R.R. (2017). Pediatric Extracorporeal Life Support Organization Registry International Report 2016. ASAIO J..

[4] Yuki K., Fujiogi M., Koutsogiannaki S. (2020). COVID-19 pathophysiology: A review. Clin. Immunol..

[5] Cascella M., Rajnik M., Cuomo A., Dulebohn S.C., Di Napoli R. (2020). Features, Evaluation and Treatment Coronavirus (COVID-19). StatPearls.

[6] Coss-Bu J.A., Klish W.J., Walding D., Stein F., Smith E.O., Jefferson L.S. (2001). Energy metabolism, nitrogen balance, and substrate utilization in critically ill children. Am. J. Clin. Nutr..

[7] Kraft R., Herndon D.N., Finnerty C.C., Hiyama Y., Jeschke M.G. (2013). Association of postburn fatty acids and triglycerides with clinical outcome in severely burned children. J. Clin. Endocrinol. Metab..

[8] Jeschke M.G., Finnerty C.C., Herndon D.N., Song J., Boehning D., Tompkins R.G., Baker H.V., Gauglitz G.G. (2012). Severe injury is associated with insulin resistance, endoplasmic reticulum stress response, and unfolded protein response. Ann. Surg..

[9] Joosten K.F., Kerklaan D., Verbruggen S.C. (2016). Nutritional support and the role of the stress response in critically ill children. Curr. Opin. Clin. Nutr. Metab. Care.

[10] Hoffer L.J., Bistrian B.R. (2013). Why critically ill patients are protein deprived. JPEN J. Parenter Enteral Nutr..

[11] Crouser E.D. (2004). Mitochondrial dysfunction in septic shock and multiple organ dysfunction syndrome. Mitochondrion.

[12] Modre-Osprian R., Osprian I., Tilg B., Schreier G., Weinberger K.M., Graber A. (2009). Dynamic simulations on the mitochondrial fatty acid beta-oxidation network. BMC Syst. Biol..

[13] Maile M.D., Standiford T.J., Engoren M.C., Stringer K.A., Jewell E.S., Rajendiran T.M., Soni T., Burant C.F. (2018). Associations of the plasma lipidome with mortality in the acute respiratory distress syndrome: A longitudinal cohort study. Respir. Res..

[14] Briassoulis G., Venkataraman S., Thompson A. (2010). Cytokines and metabolic patterns in pediatric patients with critical illness. Clin. Dev. Immunol..

[15] Eckel R.H., Grundy S.M., Zimmet P.Z. (2005). The metabolic syndrome. Lancet.

[16] Savage D.B., Petersen K.F., Shulman G.I. (2005). Mechanisms of insulin resistance in humans and possible links with inflammation. Hypertension.

[17] Steinberg D. (2004). Thematic review series: The pathogenesis of atherosclerosis. An interpretive history of the cholesterol controversy: Part I. J. Lipid Res..

[18] Steinberg D. (2005). Thematic review series: The pathogenesis of atherosclerosis. An interpretive history of the cholesterol controversy: Part II: The early evidence linking hypercholesterolemia to coronary disease in humans. J. Lipid Res..

[19] Wellen K.E., Hotamisligil G.S. (2005). Inflammation, stress, and diabetes. J. Clin. Investig..

[20] Ott J., Hiesgen C., Mayer K. (2011). Lipids in critical care medicine. Prostaglandins Leukot. Essent. Fat. Acids.

[21] Mogensen K.M., Lasky-Su J., Rogers A.J., Baron R.M., Fredenburgh L.E., Rawn J., Robinson M.K., Massarro A., Choi A.M., Christopher K.B. (2017). Metabolites Associated With Malnutrition in the Intensive Care Unit Are Also Associated With 28-Day Mortality. JPEN J. Parenter Enteral Nutr..

[22] Postle A.D. (2009). Phospholipid lipidomics in health and disease. Eur. J. Lipid Sci. Technol..

[23] Sales S., Graessler J., Ciucci S., Al-Atrib R., Vihervaara T., Schuhmann K., Kauhanen D., Sysi-Aho M., Bornstein S.R., Bickle M. (2016). Gender, Contraceptives and Individual Metabolic Predisposition Shape a Healthy Plasma Lipidome. Sci. Rep..

[24] Prokop J.W., Shankar R., Gupta R., Leimanis M.L., Nedveck D., Uhl K., Chen B., Hartog N.L., Van Veen J., Sisco J.S. (2020). Virus-induced genetics revealed by multidimensional precision medicine transcriptional workflow applicable to COVID-19. Physiol. Genom..

[25] Proulx F., Fayon M., Farrell C.A., Lacroix J., Gauthier M. (1996). Epidemiology of sepsis and multiple organ dysfunction syndrome in children. Chest.

[26] Schofield W.N. (1985). Predicting basal metabolic rate, new standards and review of previous work. Hum. Nutr. Clin. Nutr..

[27] Medicine I.O. (2006). Dietary Reference Intakes: The Essential Guide to Nutrient Requirements.

[28] Harris P.A., Taylor R., Thiekle R., Payne J., Gonzalez N., Conde J.G. (2009). Research electronic capture (REDCap)—A metadata-driven methodology and workflow process for providing translational research informatics supports. J. Biomed. Inf..

[29] Cai X., Li R. (2016). Concurrent profiling of polar metabolites and lipids in human plasma using HILIC-FTMS. Sci. Rep..

[30] Haimi P., Uphoff A., Hermansson M., Somerharju P. (2006). Software tools for analysis of mass spectrometric lipidome data. Anal. Chem..

[31] Lydic T.A., Busik J.V., Reid G.E. (2014). A monophasic extraction strategy for the simultaneous lipidome analysis of polar and nonpolar retina lipids. J. Lipid Res..

[32] Vuckovic D. (2018). Improving metabolome coverage and data quality: Advancing metabolomics and lipidomics for biomarker discovery. Chem. Commun..

[33] R Core Team (2019). R: A Language and Environment for Statistical Computing.

[34] Cribari-Neto F., Zeileis A. (2010). Beta Regression in R. J. Stat. Softw..

[35] Bates D., Maehler M., Bolker B., Walker S. (2015). Fitting Linear Mixed-Effects Models Using lme4. J. Stat. Softw..

[36] Lenth R. (2019). Emmeans: Estimated Marginal Means, aka Least-Squares Means.

[37] Zhang D. (2020). R-Squared and Related Measures 2.0.

[38] Quehenberger O., Armando A.M., Brown A.H., Milne S.B., Myers D.S., Merrill A.H., Bandyopadhyay S., Jones K.N., Kelly S., Shaner R.L. (2010). Lipidomics reveals a remarkable diversity of lipids in human plasma. J. Lipid Res..

[39] Van Dreden P., Woodhams B., Rousseau A., Dreyfus J.F., Vasse M. (2013). Contribution of procoagulant phospholipids, thrombomodulin activity and thrombin generation assays as prognostic factors in intensive care patients with septic and non-septic organ failure. Clin. Chem. Lab. Med..

[40] Arshad H., Alfonso J.C.L., Franke R., Michaelis K., Araujo L., Habib A., Zboromyrska Y., Lücke E., Strungaru E., Akmatov M.K. (2019). Decreased plasma phospholipid concentrations and increased acid sphingomyelinase activity are accurate biomarkers for community-acquired pneumonia. J. Transl. Med..

[41] Caspar-Bauguil S., Genestal M., Rajendram R., Preedy V.R., Patel V.B. (2015). Plasma Phospholipid Fatty Acid Profiles in Septic Shock. Diet and Nutrition in Critical Care.

[42] Monte M.J., Cava F., Esteller A., Jimenez R. (1989). Inhibition of biliary cholesterol and phospholipid secretion during cyclobutyrol-induced hydrocholeresis. Biochem. J..

[43] O’Donnell V.B., Rossjohn J., Wakelam M.J. (2018). Phospholipid signaling in innate immune cells. J. Clin. Investig..

[44] Dietz W., Santos-Burgoa C. (2020). Obesity and its Implications for COVID-19 Mortality. Obesity.

[45] Du Y., Tu L., Zhu P., Mu M., Wang R., Yang P., Wang X., Hu C., Ping R., Hu P. (2020). Clinical Features of 85 Fatal Cases of COVID-19 from Wuhan. A Retrospective Observational Study. Am. J. Respir. Crit. Care Med..

[46] Wu D., Shu T., Yang X., Song J.-X., Zhang M., Yao C., Liu W., Huang M., Yu Y., Yang Q. (2020). Plasma Metabolomic and Lipidomic Alterations Associated with COVID-19. Natl. Sci. Rev..

[47] Sugo T., Tachimoto H., Chikatsu T., Murakami Y., Kikukawa Y., Sato S., Kikuchi K., Nagi T., Harada M., Ogi K. (2006). Identification of a lysophosphatidylserine receptor on mast cells. Biochem. Biophys. Res. Commun..

[48] Bruni A., Mietto L., Bellini F., Ponzin D., Caselli E., Toffano G., Gorrod J.W., Albano O., Ferrari E., Papa S. (1991). Regulation of immune cells by serine phospholipids. Molecular Basis of Neurological Disorders and Their Treatment.

[49] Frasch S.C., Zemski-Berry K., Murphy R.C., Borregaard N., Henson P.M., Bratton D.L. (2007). Lysophospholipids of different classes mobilize neutrophil secretory vesicles and induce redundant signaling through G2A. J. Immunol..

[50] Frasch S.C., Berry K.Z., Fernandez-Boyanapalli R., Jin H.S., Leslie C., Henson P.M., Murphy R.C., Bratton D.L. (2008). NADPH oxidase-dependent generation of lysophosphatidylserine enhances clearance of activated and dying neutrophils via G2A. J. Biol. Chem..

[51] Frasch S.C., Fernandez-Boyanapalli R.F., Berry K.Z., Leslie C.C., Bonventre J.V., Murphy R.C., Henson P.M., Bratton D.L. (2011). Signaling via macrophage G2A enhances efferocytosis of dying neutrophils by augmentation of Rac activity. J. Biol. Chem..

[52] Hosono H., Aoki J., Nagai Y., Bandoh K., Ishida M., Taguchi R., Arai H., Inoue K. (2001). Phosphatidylserine-specific phospholipase A1 stimulates histamine release from rat peritoneal mast cells through production of 2-acyl-1-lysophosphatidylserine. J. Biol. Chem..

[53] DeCathelineau A.M., Henson P.M. (2003). The final step in programmed cell death: Phagocytes carry apoptotic cells to the grave. Essays Biochem..

[54] Bratton D.L., Henson P.M. (2011). Neutrophil clearance: When the party is over, clean-up begins. Trends Immunol..

[55] Frasch S.C., Fernandez-Boyanapalli R.F., Berry K.A., Murphy R.C., Leslie C.C., Nick J.A., Henson P.M., Bratton D.L. (2013). Neutrophils regulate tissue Neutrophilia in inflammation via the oxidant-modified lipid lysophosphatidylserine. J. Biol. Chem..

[56] Frazier W.J., Hall M.W. (2008). Immunoparalysis and adverse outcomes from critical illness. Pediatr. Clin. N. Am..

[57] Shankar R., Leimanis M.L., Newbury P.A., Liu K., Xing J., Nedveck D., Kort E.J., Prokop J.W., Zhou G., Bachmann A.S. (2020). Gene expression signatures identify paediatric patients with multiple organ dysfunction who require advanced life support in the intensive care unit. EBioMedicine.

[58] Birge R.B., Boeltz S., Kumar S., Carlson J., Wanderley J., Calianese D., Barcinski M., Brekken R.A., Huang X., Hutchins J.T. (2016). Phosphatidylserine is a global immunosuppressive signal in efferocytosis, infectious disease, and cancer. Cell Death Differ..

[59] Gervassi A.L., Horton H. (2014). Is Infant Immunity Actively Suppressed or Immature?. Virology.

