# Supplementary material for: Pediatric Multi-Organ Dysfunction Syndrome: Analysis by an Untargeted “Shotgun” Lipidomic Approach Reveals Low-Abundance Plasma Phospholipids and Dynamic Recovery over 8-Day Period, a Single-Center Observational Study
Source: Nutrients. 2021 Feb 27;13(3):774. doi: 10.3390/nu13030774 (PMC7997359; doi:10.3390/nu13030774)
Supplement: Supplementary file 1 [file nutrients-13-00774-s001.pdf]

Supplemental Table 1. Descriptive Patient Nutrition for MODS/ECMO Study 2016-2018 (*N*=24)

	Prior to admit<br>(Range) median	Baseline<br>(Range) median	72 hours<br>(Range) median	8 days<br>(Range) median
**MODS**	**(n=16)**	**(n=16)**	**(n=15)**	**(n=8)**
PO	9 (56.3)<br>6-general diet<br>1-mother's milk<br>1-Enfamil Newborn<br>1-Enfamil Infant	0 (0.0)	0 (0.0)	1 (12.5)<br>1-general diet
TF	5 (31.3)<br>1-Pediasure Fiber 1.0<br>1-Nutramigen<br>1-Pediasure Sidekicks<br>1-Pediasure Fiber 1.0 + Pediasure 1.5 Fiber<br>1-Osmolite 1 Cal	4 (25.0)<br>1-Enfamil Newborn<br>1-Nutramigen<br>1-Osmolite 1 Cal<br>1-Pediasure 1.0	9 (60.0)<br>4-Pediasure 1.0<br>1-Osmolite 1 Cal<br>1-Nutramigen<br>1-Promote<br>1-Pediasure 1.5 + Beneprotein<br>1-mother's milk	7 (87.5)<br>1-Enfalyte<br>1-Osmolite 1 Cal + Beneprotein<br>1-Pediasure Sidekicks<br>1-Pediasure Peptide1.0<br>1-Osmolite 1.5<br>1-Pediasure Fiber 1.0<br>1-mother's milk
TF+TPN/Lipids	0 (0.0)	0 (0.0)	1 (6.7)<br>TF: Enfamil Newborn<br>TPN: dextrose, TrophAmine, Intralipid	0 (0.0)
TPN/Lipids	0 (0.0)	2[a] (12.5)<br>1-Intralipid only<br>1-dextrose, Travasol, Intralipid	2 (13.3)<br>1-dextrose, TrophAmine, Intralipid<br>1-dextrose, Travasol, Intralipid	0 (0.0)
NPO	0 (0.0)	10 (62.5)	2 (13.3)	1 (0.0)
N/A	2 (12.5)	0 (0.0)	1 (6.7)	8 (0.0)
**ECMO**	**(n=8)**	**(n=8)**	**(n=7)**	**(n=6)**
PO	4 (50.0)<br>2-mother's milk<br>2-general diet	0 (0.0)	0 (0.0)	0 (0.0)
TF	3 (37.5)<br>1-mother's milk or Enfamil Infant<br>1-Elecare Jr<br>1-Enfamil Infant	2 (25.0)<br>1-mother's milk<br>1-mother's milk or Enfamil Infant	2 (28.6)<br>1-mother's milk fortified with Enfamil Infant<br>1-Elecare Jr	2 (33.3)<br>1-mother's milk<br>1-Enfamil Infant
TF+TPN/Lipids	0 (0.0)	0 (0.0)	0 (0.0)	2b (33.3)<br>1-mother's milk/dextrose, TrophAmine,

				Intralipid<br>1-Elecare Jr/dextrose, Travasol, no lipids
TPN/Lipids	0 (0.0)	0 (0.0)	3c (42.9)<br>1 dextrose, Travasol, Intralipid<br>1 dextrose, Travasol, (no lipid)<br>1 dextrose, TrophAmine, Intralipid	1 (16.7)<br>1-dextrose, TrophAmine, Intralipid
NPO	0 (0.0)	6 (75.0)	2 (28.6)	1 (16.7)
N/A	1 (12.5)	0 (0.0)	0 (0.0)	0 (0.0)

Notes: Enfamil Enfalyte, Enfamil Infant, Enfamil Newborn, Nutramigen: Mead Johnson Nutrition, Chicago, IL, USA; Elecare Jr, Osmolite 1.0, Osmolite 1.5, Pediasure 1.0, Pediasure Fiber 1.0, Pediasure Fiber 1.5, Pediasure Sidekicks, Promote: Abbott Nutrition, Columbus, OH, USA; Beneprotein: Nestle HealthCare Nutrition, Inc, Bridgewater, NJ, USA; TPN: all 70% dextrose, 10% Travasol, Baxter Healthcare, Deerfield, IL, USA; 10% TrophAmine, B Braun, Bethlehem, PA, USA; Lipids: Intralipid, Fresenius Kabi, Lake Zurich, IL, USA. All patients administered lipid emulsion received the same product, which is soybean oil, but each received differing total amounts per kilogram. The fat content of enteral products varies in percentage of total calories (47.7 – 48.0% infant, 30 – 43.7% pediatric, 23.4 – 29.5% adolescent/adult) as well as in sources of fat. Caloric and fat content of mother's milk can vary from the beginning to end of a feeding, as well as from feeding to feeding, and between mothers. None of the mother's milk was analyzed for calorie or fat content.

Supplemental Table 2. Percent calories and protein for MODS/ECMO Study 2016-2018 (*N*=24)

	Baseline		72 hr		8 days	
**MODS**	calories/% goal	gm pro/%goal	calories/%goal	gm pro/%goal	calories/%goal	gm pro/% goal
#1	0/0	0/0	44.1/12.3%	1.6/22.3%	no day 8 collection	
#2	0/0	0/0	198.7/45%	2.2/60%	no day 8 collection	
#3	0/0	0/0	132.7/20.7%	5.3/17.2%	154/24.1%	7.9/25.4%
#4	0/0	0/0	1104.3/68.1%	46.2/62.4%	1056/65.1%	44/59.5%
#5	0/0	0/0	237.9/34.6%	6.7/38.8%	no day 8 collection	
#6	0/0	0/0	104/21%	4.9/16%	234.3/47.2%	12/39.1%
#7	no data, no RD assessment					
#8	no data, no RD assessment					
#9	0/0	0/0	92/7.2%	2.8/5.5%	no day 8 collection	
#10	180/22.6%	5.4/24.5%	580.3/73%	17.4/79.1%	513.4/64.5%	15.4/65.5%
#11	0/0	0/0	211.8/ 11.5%	8.9/9.1%	440/23.9%	27/27.8%
#12	0/0	0/0	593.6/41.7%	23.7/35.2%	1158.5/81%	52/77.4%
#13	113.1/47.7%	56.6/41.6%	2169.1/91.6%	108.5/79.7%	no day 8 collection	
#14	0/0	0/0	321.3/36.5%	9.2/36.8%	222/18%	6.7/26.7
#15	20/3.9%	0.3/2.7%	168/32.9%	2.5/22.7%	107.6/21.1%	2.6/23.6%
#16	2510/104.8%	147.4/127%	2554/106%	146.1/125.9%	no day 8 collection	
**ECMO**	calories/% goal	gm pro/%goal	calories/%goal	gm pro/%goal	calories/%goal	gm pro/% goal
#1	13/0.3%	1.4/17.5%	no 72 hr collection		no day 8 collection	
#2	0/0	0/0	0/0	0/0	137.8/28.3%	7.3/69.8%
#3	341.6/76.6%	5.9/49.2%	285.3/64%	14.7/122.5%	321.2/72%	11.6/96.7%
#4	0/0	0/0	202.9/50.2%	5.2/61.9%	340.6/84.3%	6.8/81.2%
#5	0/0	0/0	60.3/9.9%	1.9/11.6%	349.2/57.2%	10.8/67.1
#6	0/0	0/0	789.1/44.7%	399/44.3%	1180.8/66.9	499/55.4%
#7	0/0	0/0	0/0	0/0	no day 8 collection	
#8	0/0	0/0	155/34.8%	4.8/51%	233.7/52.5%	5.8/59.3%

Supplemental Figures:

Lipids at the species level were explored to determine the granularity of the disease event, namely phosphotidylcholine (PC), cholesterol (chol), DG's, TG's, SM's (see Supplemental Figures S1-S5). None of the species were found to be statistically different from the sedation controls, however the heatmaps revealed qualitative differences for chol, DG's and TG's and SM's. For the PC GPC43:01 was completely absent in some MODS patients (Figure S1, A, B, C). Chol species patterns non-significantly differed from MODS as compared to control for Chol(16:0), Chol(16:1), Chol(18:3), and Chol(20:3) for both MODS and ECMO (Figure S2, A, B, C). DG's were detected in both MODS and ECMO patient, however absent in the sedation controls [DG(34:01), DG(36:01), DG(38:01) were present in all MODS sick patients, DG(36:02), DG(36:03) in MODS only and DG(37:01), DG(39:01) in ECMO only (Figure S3, A, B, C)]. DG's are highly unstable, did not resolve or model well, for this reason we were unable to include a bar chart or the 95% false coverage intervals of the mean. TG's revealed MODS patients to include many unsaturated (double bonds present) species: TG(44:01), TG(51:01), TG(53:04), TG(54:07), TG(56:02), TG(56:03), TG(56:04), TG(58:03), TG(58:04). In ECMO patients, there were generally more saturated (no double bonds) TG molecules namely, TG52:00, TG(42:00), TG(49:00), TG(50:00), TG(51:00), TG(53:00), TG(53:01), TG(54:01), TG(55:01), TG(56:01), TG(56:08), TG(57:01), TG(58:01) (Figure S4, A, B, C). SM's reveal another distinct pattern, specific for each disease trajectory, with the least SM's present in MODS patients (Figure S5 A, B, C).

Figure S1 Lipid species: Phosphotidylcholine

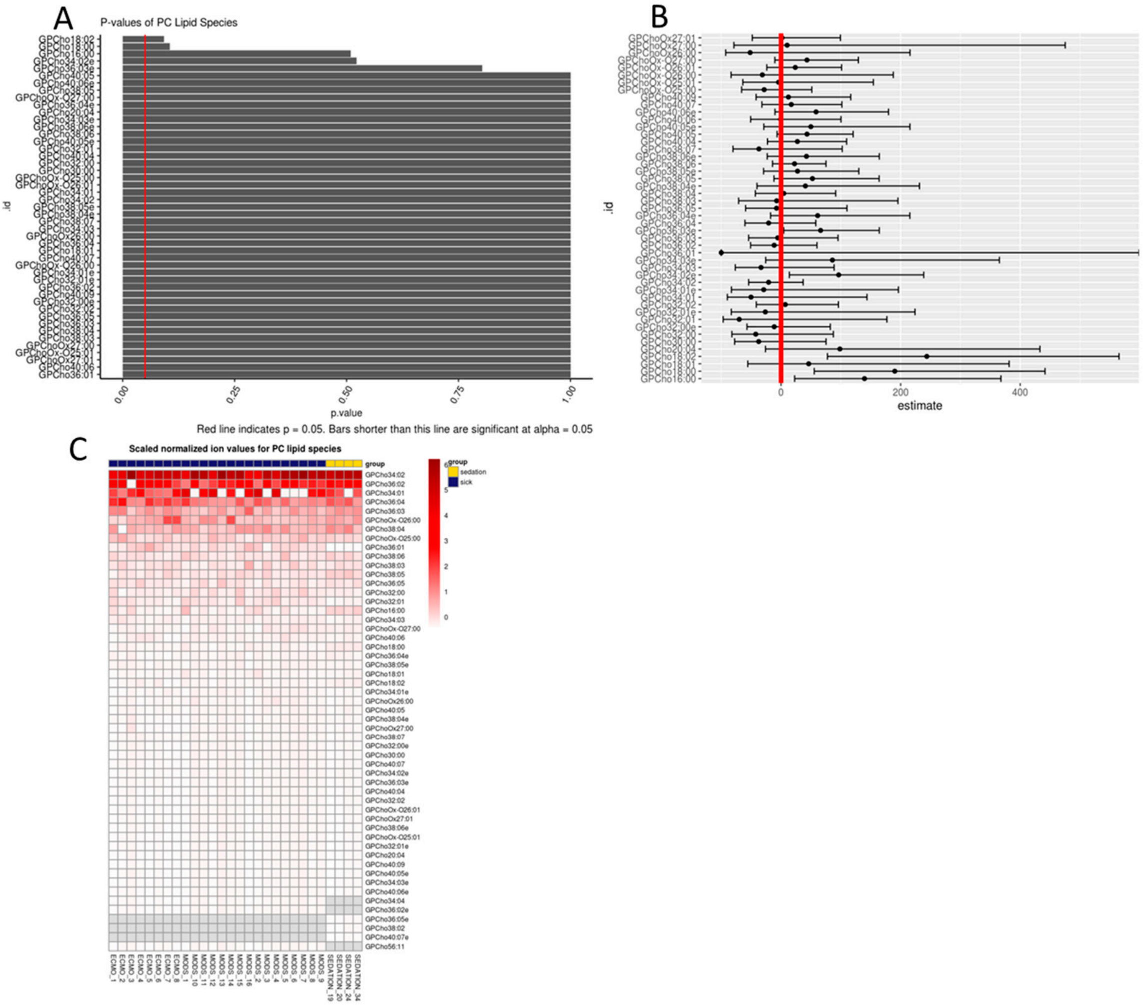

A- Bar chart of p-values from regression output from log-transformed data; red line indicates 0.05. Any p-value that crosses 0.05 is not statistically significant. B- Points in B represent the mean log(fold-change) in odds between sick and sedation groups. The error bars in B, represent the upper and lower bounds of the false-coverage for the estimate. 95% false coverage intervals of the mean log (fold-change) in odds; red line indicates 0. Any confidence interval that crosses 0 is not statistically significant. C- Scaled values of total lipids based on absolute normalized ion abundances per ml of plasma. A- Bar chart of p-values from regression output; red line indicates 0.05. Any p-value that crosses 0.05 is not statistically significant. B-Points in B represent the mean log(fold-change) in odds between sick and sedation groups. The error bars in B, represent the upper and lower bounds of the false-coverage for the estimate. 95% false coverage intervals of the mean log (fold-change) in odds; red line indicates 0. Any confidence interval that crosses 0 is not statistically significant. C- Scaled values of total lipids based on absolute normalized ion abundances per ml of plasma.

Figure S2 Lipid species: Cholesterol

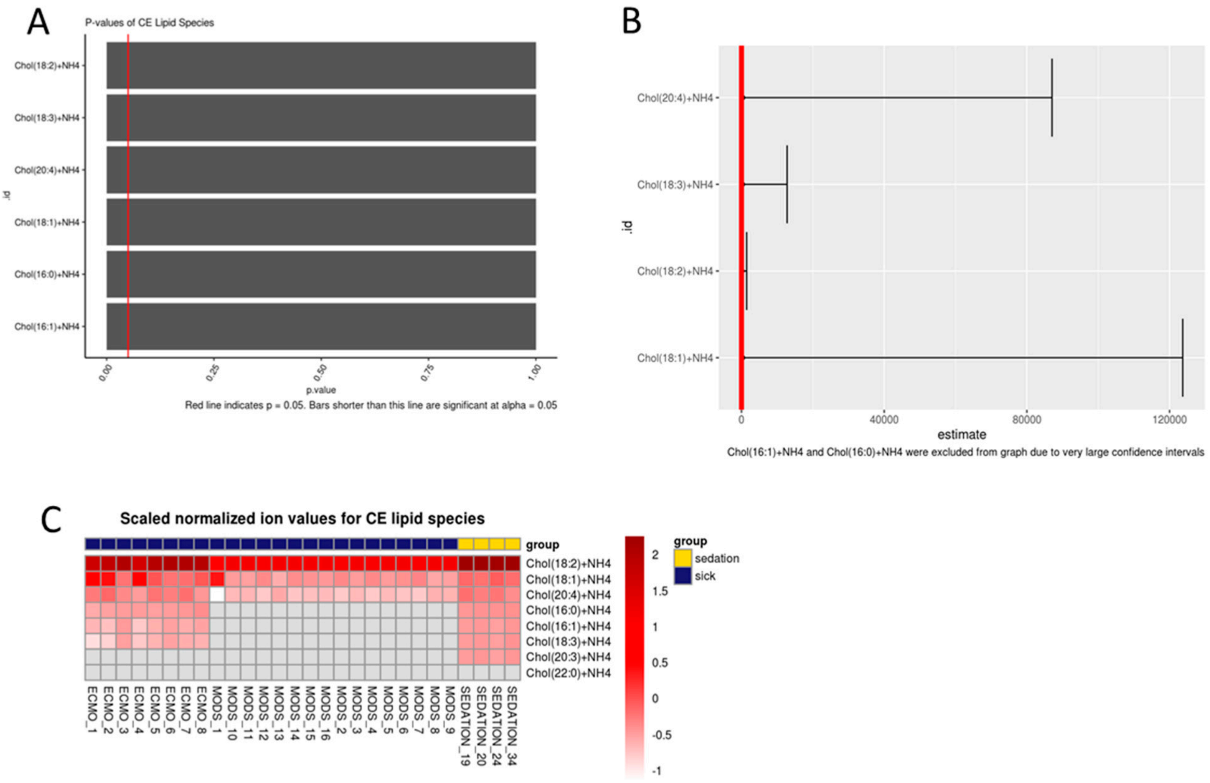

A- Bar chart of p-values from regression output from log-transformed data; red line indicates 0.05. Any p-value that crosses 0.05 is not statistically significant. B- Points in B represent the mean log(fold-change) in odds between sick and sedation groups. The error bars in B, represent the upper and lower bounds of the false-coverage for the estimate. 95% false coverage intervals of the mean log (fold-change) in odds; red line indicates 0. Any confidence interval that crosses 0 is not statistically significant. C- Scaled values of total lipids based on absolute normalized ion abundances per ml of plasma.

Figure S3 Lipid species: Diacylglycerol

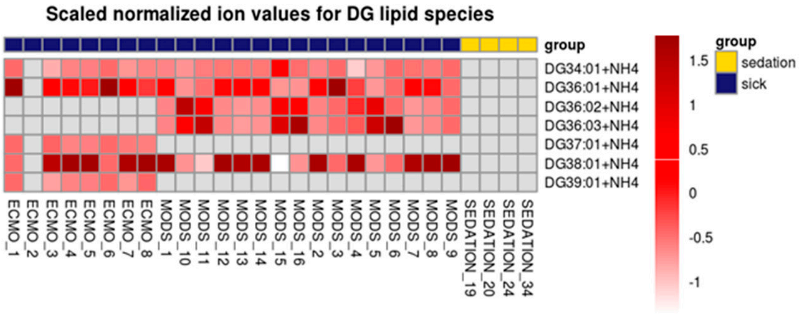

C- Scaled values of total lipids based on absolute normalized ion abundances per ml of plasma.

Figure S4 Lipid species: Triacylglycerol

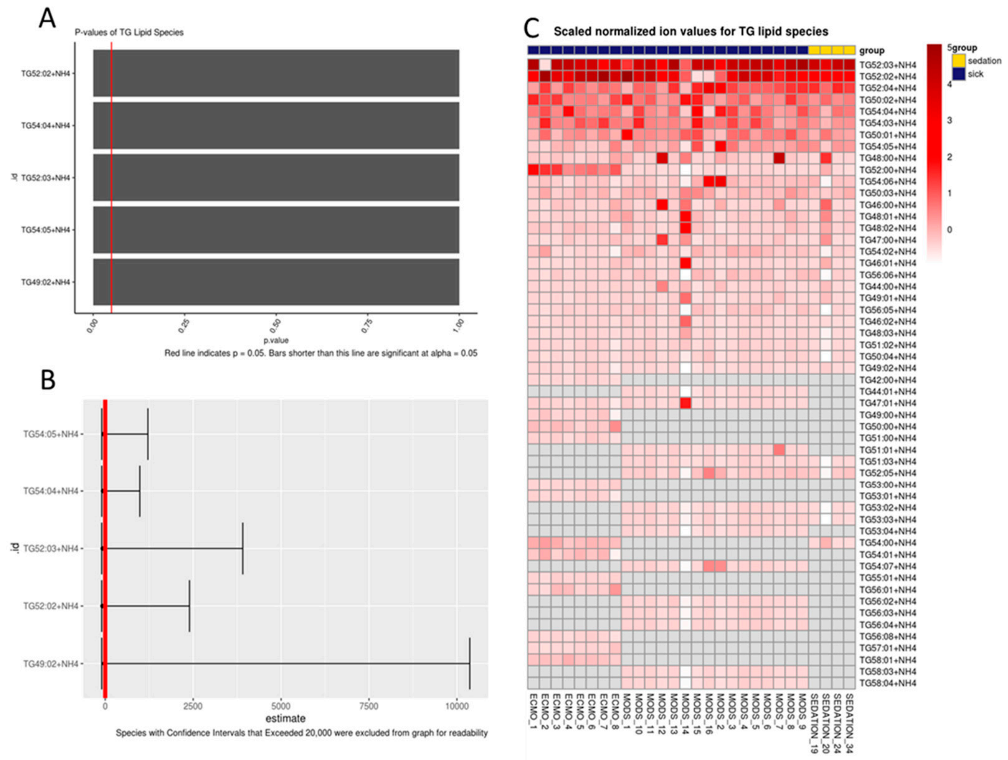

A- Bar chart of p-values from regression output from log-transformed data; red line indicates 0.05. Any p-value that crosses 0.05 is not statistically significant. B- Points in B represent the mean log(fold-change) in odds between sick and sedation groups. The error bars in B, represent the upper and lower bounds of the false-coverage for the estimate. 95% false coverage intervals of the mean log (fold-change) in odds; red line indicates 0. Any confidence interval that crosses 0 is not statistically significant. C- Scaled values of total lipids based on absolute normalized ion abundances per ml of plasma.

Figure S5 Lipid species: Sphingomyelins

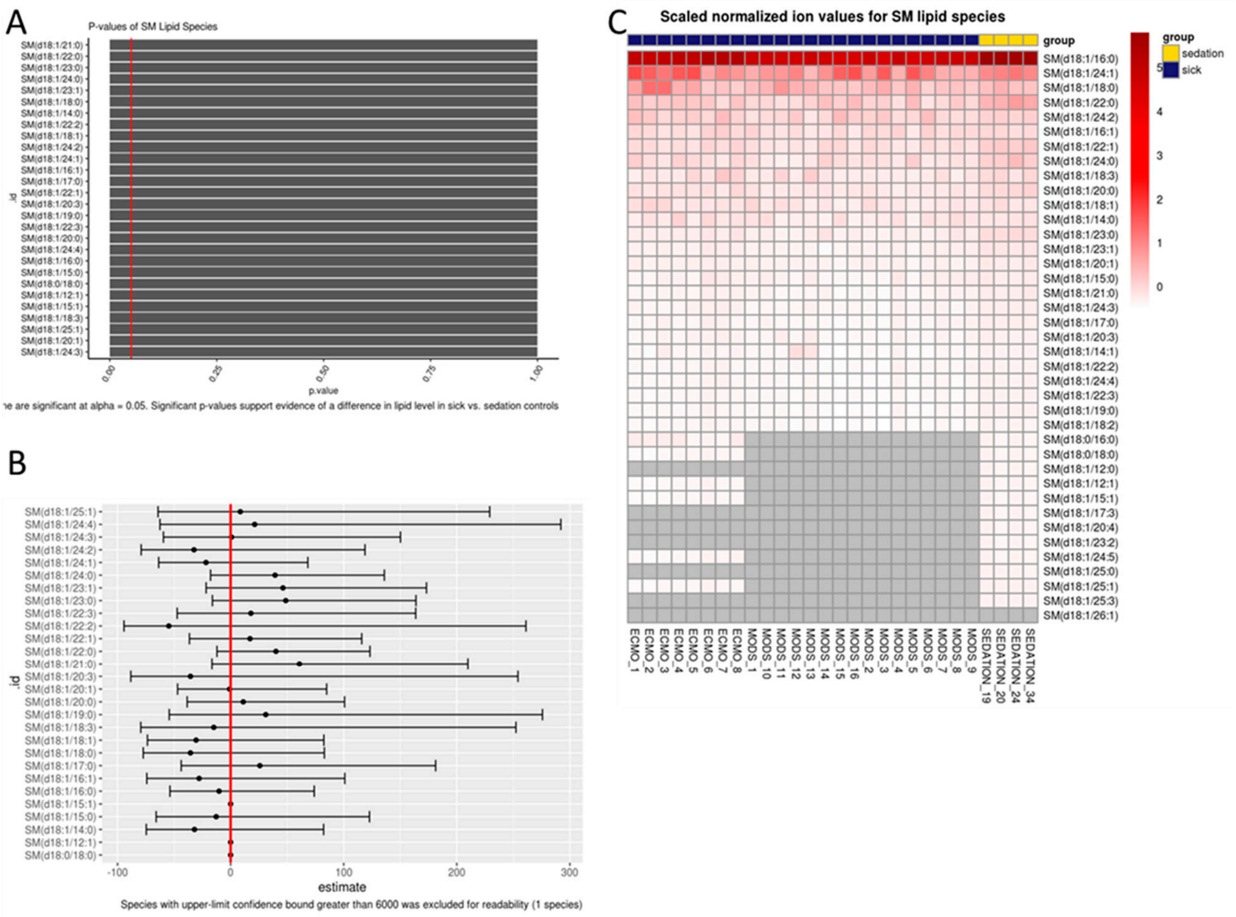

A- Bar chart of p-values from regression output from log-transformed data; red line indicates 0.05. Any p-value that crosses 0.05 is not statistically significant. B- Points in B represent the mean log(fold-change) in odds between sick and sedation groups. The error bars in B, represent the upper and lower bounds of the false-coverage for the estimate. 95% false coverage intervals of the mean log (fold-change) in odds; red line indicates 0. Any confidence interval that crosses 0 is not statistically significant. C- Scaled values of total lipids based on absolute normalized ion abundances per ml of plasma.

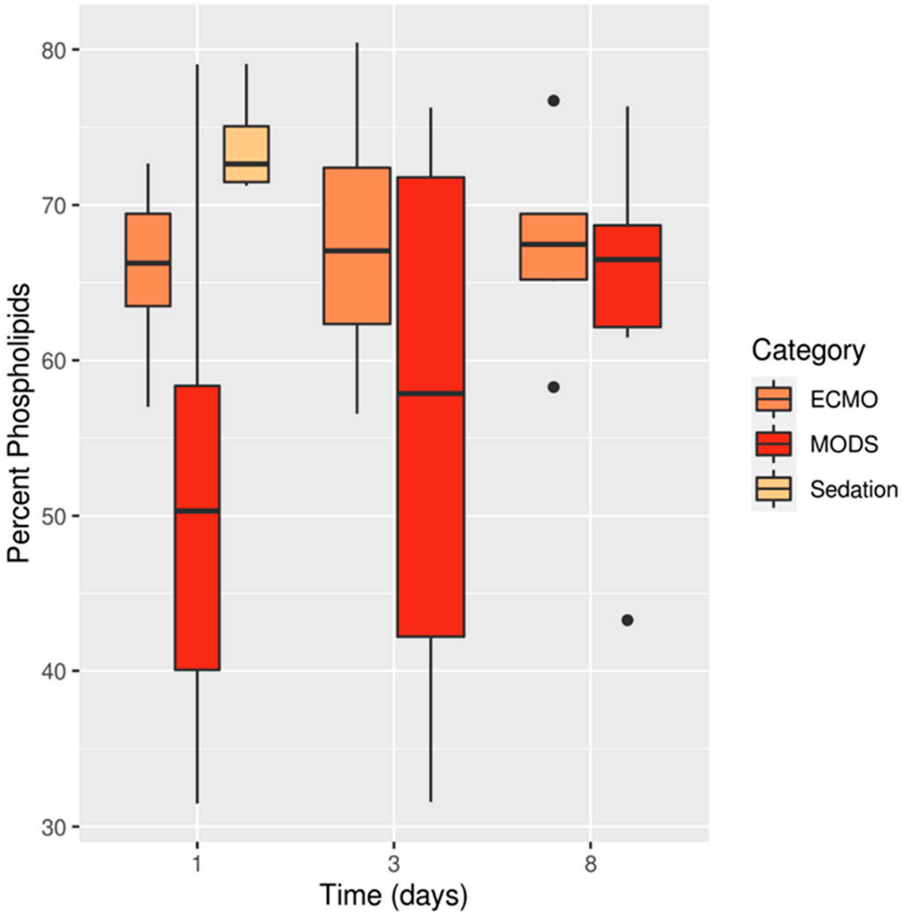

Figure S6: Boxplots of percent phospholipid values colored by group and observed at all three time points.

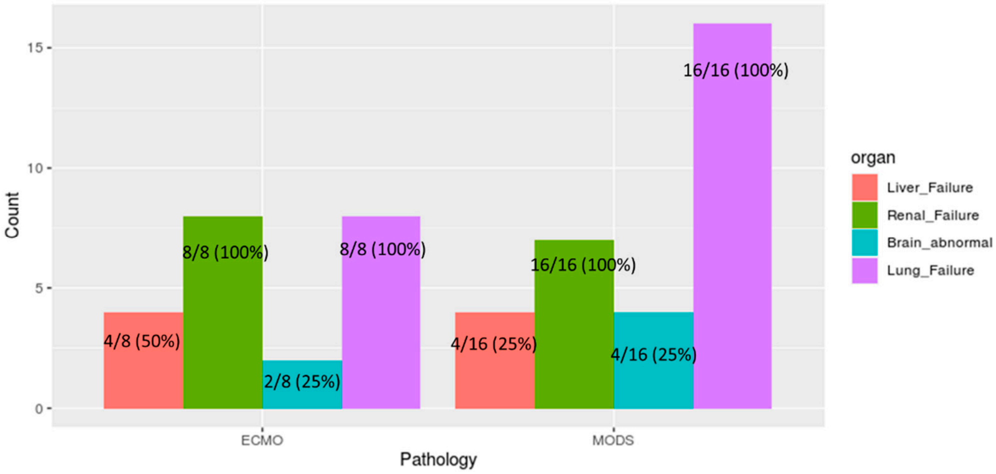

Figure S7: Organ failure by grouping. Each patient has more than one organ failing at baseline.
